# Conjugated Linoleic Acid–Carboxymethyl Chitosan Polymeric Micelles to Improve the Solubility and Oral Bioavailability of Paclitaxel

**DOI:** 10.3390/pharmaceutics16030342

**Published:** 2024-02-28

**Authors:** Iqra Mubeen, Ghulam Abbas, Shahid Shah, Abdullah A Assiri

**Affiliations:** 1 Department of Pharmaceutics, Faculty of Pharmaceutical Sciences, Government College University Faisalabad, Faisalabad 38000, Pakistan; iqramubeen1115@gmail.com; 2 Department of Pharmacy Practice, Faculty of Pharmaceutical Sciences, Government College University Faisalabad, Faisalabad 38000, Pakistan; shahid.waris555@gmail.com; 3 Department of Clinical Pharmacy, College of Pharmacy, King Khalid University, Abha 61421, Saudi Arabia; aalabdullah@kku.edu.sa

**Keywords:** linoleic acid, carboxymethyl chitosan, micelles, paclitaxel, solubility, bioavailability

## Abstract

Oral delivery, the most common method of therapeutic administration, has two significant obstacles: drug solubility and permeability. The challenges of current oral medicine delivery are being tackled through an emerging method that uses structures called polymeric micelles. In the present study, polymeric micelles were developed using conjugates of linoleic acid–carboxymethyl chitosan (LA-CMCS) for the oral delivery of paclitaxel (PCL). The developed micelles were evaluated by particle size, zeta potential, Fourier transform infrared spectroscopy (FTIR), differential scanning calorimetry (DSC), and thermogravimetric analysis (TGA). When PCL was contained within micelles, its solubility increased by almost 13.65 times (around 60 µg/mL). The micelles’ zeta potentials were −29 mV, their polydispersity indices were 0.023, and their particle diameters were 93 nm. Micelles showed PCL loading and entrapment efficiencies of 67% and 61%, respectively. The sustained release qualities of the PCL release data from micelles were good. In comparison to the pure PCL suspension, the permeability of the PCL from micelles was 2.2 times higher. The pharmacokinetic data revealed that PCL with LA-CMCS micelles had a relative bioavailability of 239.17%, which was much greater than the PCL in the suspension. The oral bioavailability of PCL was effectively increased by LA-CMCS micelles according to an in vivo study on animals. The polymer choice, maybe through improved permeability, plays an essential role when assessing oral bioavailability enhancement and solubility improvement (13.65 times). The outcomes demonstrated that PCL’s solubility and pharmacokinetics were improved in the micelles of the LA-CMCS conjugate.

## 1. Introduction

The natural antineoplastic medicine paclitaxel (PCL) was first made from the bark of Taxus brevifolia. By employing cellular microtubules, this bark is particularly effective at treating a variety of malignancies, including breast, ovarian, colon, and small and non-small lung cancer. Due to PCL’s weak solubility (<0.1 µg/mL) [1] and intestinal permeability, oral administration of the drug is difficult. Additionally, the drug exhibits local toxicity, and its previously reported oral bioavailability is less than 1% [2]. Numerous initiatives have been attempted to address these issues, e.g., different dosage forms, including liposomes, microemulsion, nanoparticles, nanocrystals, and polymeric micelles [3]. Conjugated LA-CMCS is utilised to boost the permeability of PCL through the colon while resolving the solubility issue.

Polymeric micelles have been researched for the oral administration of PCL, with some modifications like the use of biodegradable and biocompatible materials due to their admirable stability, excellent drug loading capability, and distinguished biocompatibility [4]. Micelles are essential nanocarriers for the entrapment of functional ingredients, as they increase their bioavailability and stability. Nanocarriers are formed by polymers that are lipid in nature, which form vesicles and entrap drugs inside them either chemically or physically. Due to the high stability of nanocarriers, they allow for the long circulation and accumulation of the drug at the targeted site [5]. Micelles are becoming more prevalent in oral formulations because of their benefits, including their simplicity to manufacture, high drug loading capacity (up to 30%), the tiny sizes of the particles (<200 nm), and adaptability. Theoretically, components with a critical micelle concentration (CMC) can be used to produce micelles. However, the use of ingredients with low CMCs encourages the creation of micelles because the drug loading of micelles declines as the CMC increases. Furthermore, the introduction of modified materials with a high CMC would result in an increase in the total number of materials in the micelle system.

A frequent malignant tumour of the digestive system, called colorectal carcinoma, is a leading contributor to cancer-related deaths worldwide. Despite a variety of available treatments, colorectal cancer response rates are generally poor, and recurrence rates are high. Currently, the main method of treatment for colorectal cancer is chemotherapy, despite its poor prognosis and limited efficacy. According to reports, PCL is beneficial for treating colorectal cancer. Recent research has demonstrated that PCL can influence the metabolism of glucose in triple-negative breast cancer and that PCL effectively suppresses ovarian cancer stem cells by rerouting metabolic reprogramming from glycolysis to oxidative phosphorylation.

Polymeric micelles have a particular core–shell structure and are nanosized, due to which they have gained remarkable attention in the past few years. The inner structure is hydrophobic in nature and allows for the entrapment of hydrophobic drugs, whereas the shell is hydrophilic and allows aqueous solubility and stability [6]. Micelles are made from amphiphilic polymers that can form nanometre-range vesicles [7]. Chitosan is obtained by the deacetylation of chitin; the composition of chitosan is N-acetyl-D-glucosamine and β-(1,4)-linked-D-glucosamine. Because of its multiple adaptable amino groups for ligand grafting, chitosan, a type of naturally occurring cationic polymer, has been frequently employed to create the hydrophilic outer shells of micelles. Additionally, different chitosan micelles made from polymeric materials have been suggested to boost oral drug delivery [8]. The growth of the brain and nervous system depends on LA, a necessary unsaturated fatty acid that we can only obtain through the foods we eat. The fatty acid transporter’s ligand, LA, can be grafted onto micelles to target the carrier. A fatty acid may be absorbed orally, which means that the LA present in the core of the micelles not only serves as a hydrophobic area for the loaded medication but also performs the function of attracting the transporter’s attention [9]. In the current study, we have developed polymeric micelles with low-molecular-weight CMCs conjugated with LA for the oral administration of PCL. These micelles can self-assemble when they come into contact with water and encapsulate PCL, forming polymeric micelles. A core–shell structure that results from the self-assembly of amphiphilic block copolymers in aqueous solution distinguishes polymeric micelles, which are nanoscale drug delivery vehicles. Amphiphiles function as surfactants by lowering surface tension at the air–water interface in diluted aqueous solutions where they coexist separately from other molecules.

Nano formulations for the delivery of PCL have been previously reported by many researchers [10,11,12] and shown a 10 times increase in solubility and 30% loading of PCL in nanoparticles. In the present research work, the LA-CMCS conjugate enhances the solubility and loading capacity of PCL in micelles. In this study, we attempted to formulate a LA-CMCS conjugate that has good solubility, intestinal absorption, and bioavailability, enhancing the properties of PCL. The developed micelles were characterised by FTIR, DSC, and TGA in order to obtain their chemical and thermal stabilities. The developed micelles were also checked to study the release profile of PCL in simulated gastrointestinal fluids. The pharmacokinetics of PCL-loaded micelles were assessed using albino rats and compared with PCL suspension.

## 2. Materials and Methods

### 2.1. Materials

Low-molecular-weight carboxymethyl chitosan (CMCS), paclitaxel, EDC [1-ethyl-3-(3-dimethylaminopropyl)carbodiimide hydrochloride], and NHS (N-hydroxysuccinimide) were purchased from Sigma Aldrich Gmbh Germany. Linoleic acid (LA), hydrogen peroxide 30% *w*/*v*, pancreatin, pepsin, and absolute ethanol were acquired from Merck Darmstadt Germany. Distilled water and rats were obtained from the Pharmaceutics Laboratory. A human colon carcinoma cell line, Caco-2, was obtained from the American Type Culture Collection (ATCC, Manassas, VA, USA). Rabbit polyclonal anti-human BTBD7 (ab154685; Abcam, 1:1000 dilution), rabbit poly-clonal anti-human E-cadherin (ab133597; Abcam, 1:4000 dilution), rabbit poly-clonal anti-human N-cadherin (ab76057; Abcam, 1:1000 dilution), and anti-β-actin (ab8227, Abcam, 1:3000 dilution) were purchased from Abcam, Cambridge, UK.

### 2.2. Synthesis of LA-CMCS Conjugate

The conjugate (LA-CMCS) of LA and CMCS was produced by reacting the carboxyl group of LA and primary amino groups of CMCS (low molecular weight) with the help of coupling agents (EDC/NHS). Firstly, an ethanolic solution of LA, NHS, and EDC (1:1:1 molar ratio) was developed and marked as solution 1. The reaction mixture was stirred at high speed and controlled room temperature for 30 min; high agitation is useful for activating the carboxylic acid group of LA. Afterward, solution 2 was prepared by the mixing of CMCS and hydroalcoholic mixture in a 9:1 ratio. Solution 1 was added dropwiseto solution 2 and stirred for 24 h. The resulting mixture of both solutions was cleaned by using a dialysis bag in distilled water for 72 h. Eventually, LA-CMCS conjugate was obtained after lyophilisation.

### 2.3. Characterisation of LA-CMCS Conjugate by ^1^H-NMR

To characterise the chemical structure of LA-CMCS conjugate, ^1^H-NMR spectroscopy was used. The spectra of ^1^H-NMR were noted in CDCL3 by using an NMR spectrometer (Bruker, Alpha, Germany) and tetramethylsilane as an internal standard.

### 2.4. Solubility Studies of PCL

The saturated solubility of PCL was analysed in phosphate buffer at pH 7.4, both in free form and in LA-CMCS polymeric micelles loaded with PCL [13]. To achieve supersaturation, sufficient PCL was added to 1 mL of each system, which was sonicated (Branson, Germany) for 15 min and then calibrated in a water bath (Julabo, Germany) at 100 rpm and 25 ± 0.5 °C. The medication solution was ingested in aliquots of 10 µL at regular intervals from 6 h to 48 h. An HPLC method was used to measure the PCL concentration at 240 nm after passing through 0.22 µm filter paper.

### 2.5. Formulation of Polymeric Micelles

A total of 5 mg of PCL was dissolved in 12 mL of ethanol. A solution of LA-CMCS was prepared by dissolving 20 mg of the conjugate in a 20 mL mixture of D.W and ethanol (% *v*/*v*, 98:20). Ethanolic solution of PCL was added dropwiseto the polymeric solution of LA-CMCS and sonicated with a probe sonicator (WUC-9 L, Wensar^®^, Mumbai, India) using an ice bath for 12 min. After sonication, the mixture was further processed to remove organic solvent traces and impurities of small molecules by using a vacuum (Rotavapor R-210, BUCHI Labortechnik AG, Fawil, Switzerland), and centrifugation(Sigma 3K30, SIGMA Laborzentrifugen GmbH, Osterode am Harz, Germany) of the resultant mixture occurred at 10,000 rpm for 10 min. The acquired polymeric micelles were dried and stored for further use. A Similar method was used to prepare blank micelles without the addition of PCL.

### 2.6. Critical Micelle Concentration (CMC)Measurement

The CMC for the developed polymeric micelles was measured by aslight modification of the method (capillary height method) reported by Trujillo and Schramm in 2010 [14]. The association among the meniscus, capillary rise, capillary radius, and angle of contact is used in the capillary height method (micropipette method) to calculate the solution’s surface tension. The liquid’s interfacial characteristics interact with the capillary tube’s solid walls to produce the surface tension measurement. This experiment’s methodology was adapted from Anatrace Tech Bulletin 105. A 15 µL pipette was used to determine the growing solution’s height. Nine distinct surfactant concentrations were obtained through a series of titrations utilizing stock solutions of 0.001 µM and 0.02 µM hexadecylphosphocholine. Measurements of the height of all the concentrations were taken. The average of the peak levels was plotted versus concentration during the current study, which was carried out in triplicate. The CMC was chosen as the location of the intersection of the two slopes.

### 2.7. Characterisation

#### 2.7.1. Particle Size and Zeta Potential

Using the dynamic light scattering (DLS) technique, the average micelle size and size variation in the plain and PCL-loaded LA-CMCS micelles were identified. In deionised water, a diluted colloidal solution of polymeric micelles was prepared and subjected to DLS analysis with a Zetasizer (Nano ZS, Malvern Instruments, Malvern, Worcestershire, UK). The Zetasizer was used to determine the zeta potential of micelles of the same concentration in a deionised water solution. After loading of a drug, the particle size of the micelles was determined to observe the effect of drug loading on the size of the micelles. The prepared micelles were lyophilised, and the particle size of all the prepared formulations was determined by being dispersed in deionised water and compared with the size of the micelles before and after drug loading.

#### 2.7.2. Fourier Transforms Infrared Spectroscopy (FTIR)

The FTIR spectra of LA, CMCS, LA-CMCS conjugate, and unloaded and PCL-loaded micelles were obtained between 4000 and 400 cm^−1^ using an ATR-FTIR spectrometer (Bruker-Alpha, Ettlingen, Germany).

#### 2.7.3. DSC and TGA Analysis

Temperature and heat flow enthalpy were used to calibrate DSC. Baseline calibrations were carried out as advised by the manufacturer. As a reference standard, a sample of indium was used; this was analysed, melted, we noted the onset of melting and heat of fusion, and we compared these results to the known values (indium melts at 156.6 °C and its heat of fusion is 28.71 J/g, respectively). The DSC apparatus functions in an atmosphere of nitrogen and is free of contaminants. We chose the calibration mode, applied the reference material (5 mg of indium), and compared the findings to the published values. The calibration materials were heated through a recognised thermal transition area at a regulated rate in a controlled atmosphere as part of this test process. The temperature difference between the calibration material and the reference, or the heat flow into the calibration material, was continually observed and recorded. When the specimen absorbed energy, the heating curve displayed a similar endothermic peak, signifying a transition.

A thermal analyser (DSC, STA 449C thermal analysers, NETZSCH, Gerätebau, Germany) was used to conduct a DSC and TGA study. The micelle suspension was freeze-dried, and the test sample was lyophilised powder. For DCS and TGA analysis, 5 ± 0.5 mg samples of PCL, blank, and PCL-loaded micelles were placed in aluminium crucibles, heated at a temperature range of 30–500 °C with a rate of 10 °C/min, and passed through a stream flow of nitrogen gas at 40 mL/min.

#### 2.7.4. Transmission Electron Microscopy (TEM)

On a copper grid covered with carbon film, LA-CMCS2 micelles containing 0.01% phosphotungstic acid were added, and the grid was dried at 20 °C. The observation was performed using JEM-2000 FX II (Jeol, Akishima, Tokyo, Japan) at 80 kV.

### 2.8. Drug Loading and Entrapment Efficiency

The amount of drug entrapped in the formulation was determined using HPLC (Waters 2695 Separations Module) and the analytical column Phenomenex^®^ C-18 (2504.60 mm, 5 m). For PCL, the PDA detector (Waters 2696 photodiode array detector) was set to 230 nm. The mobile phase, acetonitrile, and ammonium acetate buffer (65:35)were pumped at 1.2 mL/min in isocratic mode. To summarise, 100 µL of micelles was accurately collected, and the volume was increased to 1 mL using acetonitrile to analyse the drug content via HPLC [15]. The following formulas were used to calculate the entrapment efficiency (EE) and drug loading (DL) of PCL.

(1) $$EE\left(\%\right)=\frac{Weight\;of\;PCL\;in\;micelles}{Weight\;of\;PCL\;initially\;added}\times 100$$

(2) $$DL\left(\%\right)=\frac{Weight\;of\;PCL\;in\;micelles}{Weight\;of\;micelles}\times 100$$

### 2.9. In Vitro Release Studies

In vitro release studies of PCL from the PCL-loaded LA-CMCS were analysed usinga previously reported method (dialysis bag method) with modifications [16]. A total of 2 mL solution of PCL-loaded LA-CMCS micelles containing 200 µg PCL was added into a dialysis bag (MWCO of 14 kDa) and immersed into 150 mL of each release medium, i.e., simulated gastric fluid (pH 1.2) with and without pepsin and simulated intestinal fluid (pH 6.8) with and without pancreatin. All these release tests were conducted in a water bath shaker (EQUITRON Medica Instrument Mfg. Co., Mumbai, Maharashtra, India) at 37 ± 0.5 °C shaken horizontally at 100 rpm. Then, 1 mL aliquot was withdrawn at 0.5, 1, 2, 4, 6, 8, 12, 24, 48, and 72 h and replaced with fresh medium of equal quantity to maintain sink conditions. The drug content in the withdrawn medium was assessed by the HPLC method. After dissolution, the sizes of the micelles of all the formulations were determined using the DLS method. Different kinetic models (zero and first order) along with the Korsmeyer–Peppas approach were employed to evaluate the release of PCL from the five formulations of micelles with the goal of comprehending the PCL release profiles and forecasting in vivo efficacy.

### 2.10. Stability of Micelles in Gastrointestinal Fluids

The stability of PCL-loaded LA-CMCS micelles was evaluated by using different physiological media, i.e., simulated/intimated intestinal fluid at 6.8 pH with or without the addition of pancreatin and simulated/intimated gastric fluid at 1.2 pH with or without the addition of pepsin [17]. These micelles were also assessed in PBS at pH 5.5. In all these mediums, 1 mL of solution containing 200 µg of conjugate was incubated for 6 h in simulated intestinal fluid and 2 h in simulated gastric fluid and in PBS. The samples were analysed for particle size, PDI, and surface charge after incubation.

### 2.11. Storage Stability of Micelles

The drug content and micelle size were evaluated after 4 weeks of incubation at 4 °C to evaluate the micelles (LA-CMCS1 to LA-CMCS5 formulations) storage stability in PBS. For a duration of 24 h, the micelles were incubated in PBS containing 50% foetal bovine serum at 37 °C to assess their stability in serum. The size of the micelles was observed by DLS. All measurements were made in triplicate.

### 2.12. Intestinal Permeability

The permeation of PCL from LA-CMCS micelles was measured using the in situ intestinal perfusion technique. Before the trials, rats were deprived for 12 h and allowed unrestricted access to water. A tiny section of the intestinal tract was selected as soon as the abdomen was exposed and washed with saline solution. A flow pump was used to collect the contents following the delivery of a 20% (*w*/*v*) urethane injection at a dose of 1 g/kg. A pledget drenched in saline solution was used to cover the surgical area, and a heating light was employed to maintain the rat’s internal temperature within normal range. For 10 min, Krebs–Ringer buffer (KRB) was pushed through the portion of the intestine at a flow rate of 0.2 mL/min. The drug perfusion liquid was then infused at a rate of 0.2 mL/min, and the perfusion process began.

The perfusion liquid for each of the samples evaluated in KRB contained 20 µg/mL phenol red and 30 µg/mL PCL. The average length of each part of the intestines was taken into account in addition to the perfused specimens, which were collected every 15 min up to 120 min after steady-state was reached. After centrifuging the mixture for 10 min at 10,000 rpm, we added 0.8 mL of methanol and 0.2 mL of perfusate and performed a PCL analysis on the supernatant using HPLC. Using a UV spectrophotometer and 0.1 mL of perfusate and 0.9 mL of sodium hydroxide (0.1 M), the amount of phenol red at 558 nm was measured. The equation was then used to determine the efficient permeation. In Equation (3), Q is the perfusate flow rate (0.2 mL/min), r is the intestine’s radius (0.17 cm), L is the intestinal segment’s length in centimetres, C_in_ and C_out_ are the PCL inlet and outlet concentration of perfusate, and PR_in_ and PR_out_ are the PCL in and out phenol red concentration, respectively.

(3) $$Effective\;permeability=-\frac{Q}{2\pi rL}In(\frac{C_{out}}{C_{in}}\times \frac{{PR}_{in}}{{PR}_{out}})$$

### 2.13. Biological Studies

#### 2.13.1. Cell Viability Assay

Using an MTT assay (Promega, Woods Hollow Road, Madison, WI, USA), the cytotoxic effect of the prepared micelles was evaluated. In the Caco-2 cell line, the percentage of cell viability following treatment with LA-CMCS2 (drug-loaded) was compared to that of the control, LA-CMCS2 Blank, and PCL. All cells (1 × 10^5^/well) seeded in 96-well plates were incubated for 6 h and 24 h and treated with LA-CMCS2 (drug-loaded), LA-CMCS2 blank, and PCL. An automated microplate reader was used to measure absorbance at 570 nm following MTT treatment. The ratio of cells that received therapy was compared to the controls that were calculated using the formula below.

(4) $$Cell\;viability\left(\%\right)=\frac{A570(sample)-A570(blank)}{A570(control)-A570(blank)}\times 100$$

#### 2.13.2. Haemolysis Test

This study evaluated the effects of LA-CMCS2 PCL-loaded polymeric micelles on sheep’s red blood cells (RBCs) using common haemolysis tests. To do this, a 2.5 mL suspension of RBCs was combined with various amounts of LA-CMCS2 to make a 5 mL solution. Different concentrations of LA-CMCS2 micelles were mixed with 5 mL of water based on the 2.5 mL total volume of the RBCs solution. The positive control group received deionised water, whereas the negative control group received saline solution. All the aforementioned suspensions were incubated for 1 h at 37 °C and then centrifuged for 10 min at 3000 rpm to remove intact RBCs. The collected supernatant was examined using a UV-2450 spectrophotometer (Shimadzu) to measure haemoglobin discharge at 541 nm. The % haemolysis was evaluated by using Equation (5). The cloudy appearance of the specimens was reduced by using a blank control preparation free of RBCs.

(5) $$Hemolysis\;\left(\%\right)=\frac{Samples\;absorbance-Absorbance\;of\;0\%\;hemolysis\;solution}{Absorbance\;of\;100\%\;hemolysis\;solution-Absorbance\;of\;0\%\;hemolysis\;solution}\times 100$$

#### 2.13.3. Quantitative Real-Time Polymerase Chain Reaction (qRT-PCR)

For measuring the expression of genes like E-cadherin, Slug, Vimentin, and N-cadherin, qRT-PCR was used as well as the TRIzol technique, and total RNA was isolated from cultured Caco-2 cells and quantified using a NanoDrop spectrophotometer that measured absorbance at 260 and 280 nm. Using a Thermo Scientific (Waltham, MA, USA) cDNA kit, RNA samples were converted to cDNA. As a housekeeping gene, β-actin was employed. Primers (E-cadherin, Slug, Vimentin, N-cadherin, and β-actin) were created using primer blast and the Gene Bank. qRT-PCR was used for the thermal cycling of 40 cycles, the denaturation of each cycle was at 95 °C for 15 s, annealing occurred for 20 s at 60 °C, and extension took place for 20 s at 72 °C. Mesa-Blue qPCR Master Mix-Plus for the SYBR assay (Eurogentec) and the Master cycler Realplex2 (Eppendorf) were used in qRT-PCR to evaluate the mRNA expression levels of the genes. Using cycle threshold (Ct) values, the amount of the increased product of PCR with respect to the reference gene(β-actin) was estimated.

#### 2.13.4. Western Blot Analysis

To lyse Caco-2 cells, we used RIPA lysis buffer (Beyotime, Haimen, Jiangsu, China). Using a BCA Protein Assay Kit (TaKaRa), the concentrations of total protein were determined. Proteins were separated on 10% SDS-PAGE gels and then electrophoresed before being transferred to PVDF membranes. After that, 5% BSA was used to block the membranes. After blocking, membranes were incubated with rabbit polyclonal anti-human BTBD7 (ab154685; Abcam, 1:1000 dilution), rabbit poly-clonal anti-human E-cadherin (ab133597; Abcam, 1:4000 dilution), and rabbit polyclonal anti-human N-cadherin (ab76057; Abcam, 1:1000 dilution), and anti-*β*-actin (ab8227, Abcam, 1:3000 dilution)was used as the loading control. The appropriate secondary antibodies were then added to each membrane and incubated. Lastly, we used animproved chemiluminescence kit to envisage bands of protein in a ChemiDoc XRS Plus luminescence image analyser(Bio-Rad, Hercules, CA, USA). Relative grayscale values are displayed for the results.

#### 2.13.5. Pharmacokinetic Study

Two groups of 12 albino rats (200–400 g) were selected to evaluate the pharmacokinetics of PCL-loaded polymeric micelles (LA-CMCS2). The ethical committee of Government College University Faisalabad, Faisalabad, granted permission to study with ethical approval number IRB/GCUF/0089, and the ICH guidelines were followed. On the first day, the rats fasted for 24 h. The rats in groups 1 and 2 received 10 mg/kg of the PCL suspension (reference) and the LA-CMCS2 micelles formulation (test), respectively. A total of 0.25 mL of blood was drawn from the rat tail vein at specified times (0, 1, 2, 4, 6, 8, 10, 12, 24, 36, 48, 60, and 72 h). The blood samples were promptly centrifuged at 6500 rpm for 5 min to yield samples of plasma (100 µL), which were then kept at −20 °C until HPLC analysis. T_max_, C_max_, AUMC, MRT, and AUC were estimated by employing Kinetica-R-version 4.1.1 by ThermoElectron Corporation, Waltham, MA, USA.

#### 2.13.6. Tissue Distribution Study

With minor adjustments, the blood and tissue samples were prepared according to the method outlined by Hong et al. [18] to ascertain the paclitaxel concentration. As an internal reference, 800 µL of acetonitrile containing 10 µL of 100 µg/mL P-hydroxybenzoic acid n-butyl ester was combined with 200 µL of blood samples. Following vortex mixing, the mixture was centrifuged at 13,000 rpm for 10 min. After being combined with 500 µL of distilled water, the 500 µL supernatant fraction was filtered using a 0.2 µm PTFE syringe filter (Whatman, Inc. Bridgewell Place Clifton, NJ, USA). The liver, spleen, kidney, heart, lung, stomach, intestine, and contents of the intestine were also taken out. Using a high-speed homogeniser (T-25-Ultra-Turrax; Janke & Kunkel GmbH & Co., Staufen, Germany), tissues were homogenised in five volumes of acetonitrile. After homogenizing the tissues (990 µL), 10 µL of internal standard solution was added, and the mixture was centrifuged for 30 min at 13,000 rpm. A 0.2 µm PTFE syringe filter was used to filter 400 µL of supernatant fraction after it had been combined with 600 µL of distilled water. The analysis method used for the acetonitrile extracts of blood and tissues was high-performance liquid chromatography.

## 3. Results and Discussion

### 3.1. Synthesis and Characterisation of LA-CMCS Conjugate

**Figure 1:**
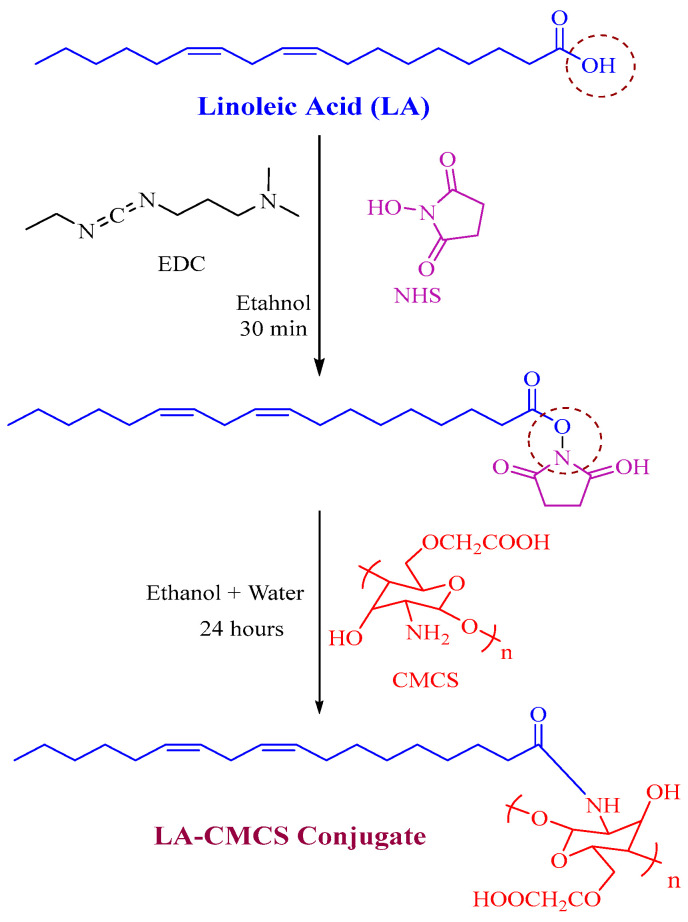
Chemical scheme for the synthesis of LA-CMCS conjugate in the presence of coupling agents (EDC and NHS).

Figure 1 provides a summary of the LA-CMCS conjugate’s thorough synthesis process. By forming an amide connection between the carboxyl groups of LA and the amine group of CMCS, the LA-CMCS conjugate was created. There is only one possibility that one mole of LA is conjugated with one mole of CMCS. Using ^1^H-NMR, the chemical structure of a freshly synthesised conjugate was examined. The H-NMR of LA, CMCS, and LACMCS conjugate is shown in Figure S1. Figure S1A illustrates the range of the olefinic protons of double bonds with conjugation and the glycerol component in LA, which are 5.30–5.46 ppm and 2.58 ppm, respectively. The ranges of protons CH_2_-CH=CH and -CH_2_ are 2.04–2.21 ppm and 1.48 ppm, respectively. In the ranges of 1.31–1.26 ppm and 0.85–0.92 ppm, respectively, are the (CH_2_)n and CH_3_ protons. According to Figure S1B, the CMCS ^1^H-NMR spectrum displays signals in the range of 3.73 to 3.62 ppm [19], which are caused by two hydrogens of the carboxymethyl group attached to C_6_ and one hydrogen of the carboxymethyl group bonded to C_3_. The signals assigned to the two hydrogens from the carboxymethyl group linked to their nitrogen group in the range of 3.03–3.15 ppm. In LA-CMCS conjugate, a peak was observed at 7.35 ppm [20], indicating the successful conjugation of LA with CMCS in the presence of coupling agents, as shown in Figure S1C.

### 3.2. Solubility Studies

The solubility of free PCL or PCL-loaded LA-CMCS2 micelles in water was investigated. PCL was barely soluble in water (4.4 µg/mL). It was found that the PCL-loaded micelles increased the solubility of PCL by approximately 13.65 times (around 60 µg/mL). Khalid et al., in 2023, also studied the solubility of PCL using disulfide bridged nanoparticles, which resulted in an 11 times enhancement of solubility, which was less compared to the present study [13].

### 3.3. Formulation of Polymeric Micelles and Critical Micelle Concentration Measurement

In an aqueous solution, the produced amphiphilic copolymers self-assembled to create core–shell structured micelles. The amphiphilic copolymers were dissolved in ethanol, a solvent compatible with both blocks, to enable LA-CMCS conjugate-based micelle formation. This was followed by the addition of distilled water and dialysis to completely remove any remaining ethanol. The surface tension of three varying amounts of cavitand (benzimidazole), 0.1 µM, 0.5 µM, and 1 µM, was measured in the initial studies using the filter paper method. The CMC occurred at 11 µM in the absence of additives, which is consistent with previous investigations that found the hexadecylphosphocholine CMC to occur between 6 µM and 13 µM. The studies carried out with the inclusion of cavitand did not, however, show a discernible CMC change. In order to ensure that there were enough data points regarding the hexadecylphosphocholine CMC values in the literature, some changes were made to the hexadecylphosphocholine concentrations. Additionally, the cavitand concentration was raised to 50 µM. The capillary height approach was used to repeat the experiment. The CMC of hexadecylphosphocholine for the capillary height method was found to be at 18.3 µM without the addition of cavitand, which is comparable with values in the literature. In the filter paper approach, the CMC increased to 32.8 µM with the addition of 50 µM of cavitand. The lower CMC value of the LA-CMCS conjugate suggests that micelles form at low concentrations and remain stable even in greatly diluted environments.

### 3.4. Characterisation

#### 3.4.1. Particle Size and Zeta Potential

Dynamic light scattering was used to estimate the hydrodynamic diameter of several micelles (DLS). The diameter of LA-CMCS1 to LA-CMCS5 before the loading of the drug ranged from 93 nm to 119 nm, as shown in Table 1. The size of the micelles of LA-CMCS2 and surface charge is outlined in Figure S2. After the loading of the drug, a slight increase in the particle size of LA-CMCS1, LA-CMCS2, and LA-CMCS4 occurred. The particle size of LA-CMCS3 was decreased, and the size of LA-CMCS5 remained the same after the loading of the drug. Micelles’ respective zeta potentials ranged from −16 mV to −29 mV, as shown in Figure S3. The usage of LA-CMCS conjugates for micelle production was attributed to the shift in particle size and zeta potential [21]. Sharma et al. noted some related phenomena, which they explained by a decrease in the aggregation number [22]. Man et al., however, found that the size of micelles increased after drug loading [23]. However, another investigation found that medication loading had no appreciable impact on micellar size [24]. In a different investigation, we further found that adding dasatinib to polymeric micelles did not appreciably alter their size [25]. There is no widespread agreement on how drug loading affects the size of polymeric micelles. After lyophilisation, a small change in micelles size was observed, as shown in Table 1.

#### 3.4.2. Fourier Transforms Infrared Spectroscopy (FTIR)

**Figure 2:**
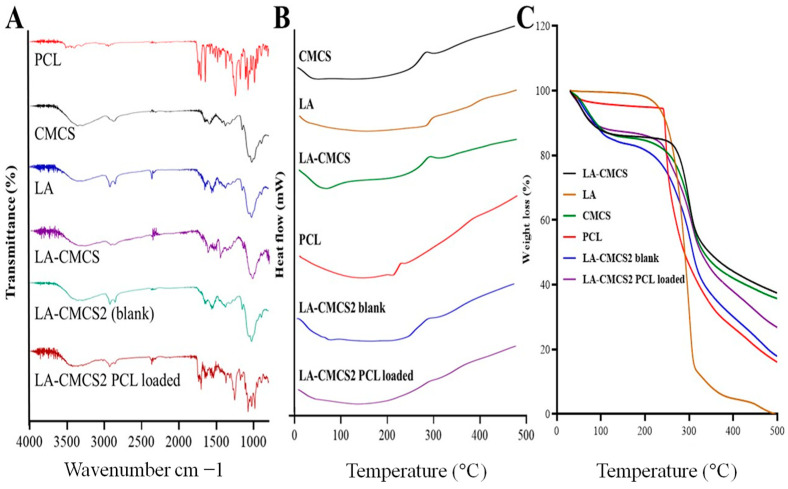
FTIR spectra (**A**), DSC thermograms (**B**), and TGA (**C**) of PCL, CMCS, LA, LA-CMCS conjugate, blank, and PCL-loaded micelles formulation LA-CMCS2.

To identify the groups of molecules involved in the reaction, we used FTIR to examine the molecular specifics of how medications react with the micelles. The functional groups -OH, C-O-C, C-C, C-N, and C-O, respectively, were assigned to the observed peaks in the FTIR spectra of PCL at wave numbers of 3619 cm^−1^, 1754 cm^−1^, 1678 cm^−1^, 1239 cm^−1^, and 1109 cm^−1^, as shown in Figure 2A. PCL observed the C-H absorbance bands in the benzene ring at 3095 cm^−1^, 770 cm^−1^, and 708 cm^−1^ [26]. CMCS showed characteristic bands at 3200–3600 cm^−1^, assigned to O-H and N-H stretching, where O-H stretching vibration was superimposed with N-H stretching. The band at 890–1100 cm^−1^ was attributed to ether groups of the CMCS saccharine structures (C-O-C stretching) [27]. The aliphatic C-H group may be responsible for the distinctive bands of LA’s FTIR that demonstrate stretching at 2870 cm^−1^ and 2810 cm^−1^. Stretching of the CO and C=O bands was seen at 1758 cm^−1^ and 1235 cm^−1^, respectively. Due to the vibration of the aliphatic C-H group, a band was seen at 782 cm^−1^. The presence of the methylene group at 1509 cm^−1^ caused the C-H group to bend and scissor [28]. The LA-CMCS conjugate had a stronger absorption peak at 1635 cm^−1^ due to the carbonyl stretching vibration of secondary amide, while the peaks at 1498 cm^−1^ and 1387 cm^−1^ were assigned to the bending vibrations of amide band II and amide band III, respectively. The results indicated that an amide linkage between -COOH of LA and -NH2 of CMCS formed in LA-CMCS, and LA was successfully conjugated to CMCS. In the FTIR spectrum of blank LA-CMCS2 micelles, the primary peaks of LA-CMCS conjugate were seen. The FTIR spectra of PCL-loaded LA-CMCS micelles (LA-CMCS2) revealed the primary functional groups of LA-CMCS and PCL. The LA-CMCS conjugate and PCL in the synthesised micelles exhibited no chemical interactions.

#### 3.4.3. DSC and TGA Analysis

The PCL exhibited two exothermic peaks, the first being at 249 °C (ΔH = 119.79 J/g) and the subsequent one at 227 °C. According to Figure 2B, these peaks demonstrate the drug’s crystalline form [29]. CMCS displayed endothermic and exothermic peaks at 65 °C (ΔH = 77.902 J/g)and 303 °C, respectively [30]. LA displayed an endothermic peak at 302 °C (ΔH = 56.852 J/g). The LA-CMCS conjugate displayed two thermal peaks, one exothermic at 304 °C (ΔH = 59.87 J/g)and the other endothermic at 77 °C [31]. The melting curve of the LA-CMCS2 blank micelles showed an exothermic peak at 310 °C, but an endothermic peak was also observed at 87.22 °C (ΔH = 24.223 J/g). The DSC curve of PCL-loaded LA-CMCS2 micelles presents an exothermic peak at 305 °C, whereas the peak of PCL completely disappears, which indicates that PCL was successfully loaded in the core of the micelles of LA-CMCS2. The deterioration and thermal resistance of the polymeric conjugate and micelles can be estimated by TGA. The graph shows that the initial stage of the disintegration of LA-CMCS takes place at higher temperatures than CMCS (34–244 °C and 34–279 °C for CMCS and LA-CMCS, respectively) and that LA-CMCS loses less weight overall than CMCS (8% and 19%, respectively) [32]. This shows that CMCS can absorb more water than LA-CMCS, both bound and unbound. The dehydration of the sugar ring and the breakdown of the polymer units may be involved in a complicated process during the second stage of decomposition. According to Figure 2C, structural disintegration occurs at temperatures between 240 and 320 °C for CMCS and 280 and 320 °C for LA-CMCS, resulting in weight reductions of 37% and 33%, respectively. Because LA-CMCS contains additional substituent groups, it is less thermally stable than CMCS and experiences a second drop in the curve at lower temperatures. Weight loss in the third stage is sluggish for both CMCS and LA-CMCS, with CMCS occurring between 320 and 500 °C and LA-CMCS between 318 and 500 °C; this may be related to CMCS deacetylation or LA breakdown. In conclusion, the weight loss of LA-CMCS under heating is slightly slower than that of CMCS.

#### 3.4.4. Transmission Electron Microscopy (TEM)

Figure S4 displays the TEM image of the LA-CMCS2 micelle. The micelles appear to have spherical forms based on the TEM data. However, the micelle diameter determined by DLS was found to be 93 nm, which is larger than that of the micelle diameter determined by TEM. This is because, in contrast to the TEM pictures, which show the micelles in their dry condition, the DLS represents the size of the hydrated micelles [33].

### 3.5. Drug Loading and Entrapment Efficiency

Drug loading (%DL) and % encapsulation efficiency (%EE) are important factors for delivering hydrophobic medicines. Table 2 displays the %EE and %DL of the PCL-loaded LA-CMCS micelle as determined by HPLC. The amount of drug entrapped in the micellar system was changed by the feed concentration ratio of the drug to the polymer. The PCL is entrapped in the synthesised micelles via the hydrophobic interaction between the drugs and the material. Together with an increase in the weight ratio of PCL to LA-CMCS from 1:1 to 1:8, the encapsulation efficiency of LA-CMCS micelles loaded with PCL decreased from 67% to 62%. The effectiveness of encapsulation was not boosted by further raising the drug feeding ratio (1:4 to 1:8). When PCL was fed at a ratio of 1:1 to the LA-CMCS polymer, the highest %EE and %DL were seen. The %EE of PCL ranged from 56 to 60%, as shown in Table 2. This LA-CMCS conjugate showed more loading of PCL compared to the nanoformulation of sodium alginate and eudragit conjugate already reported by Khalid et al. in 2023 [13].

### 3.6. Release Studies and Kinetics of PCL

**Figure 3:**
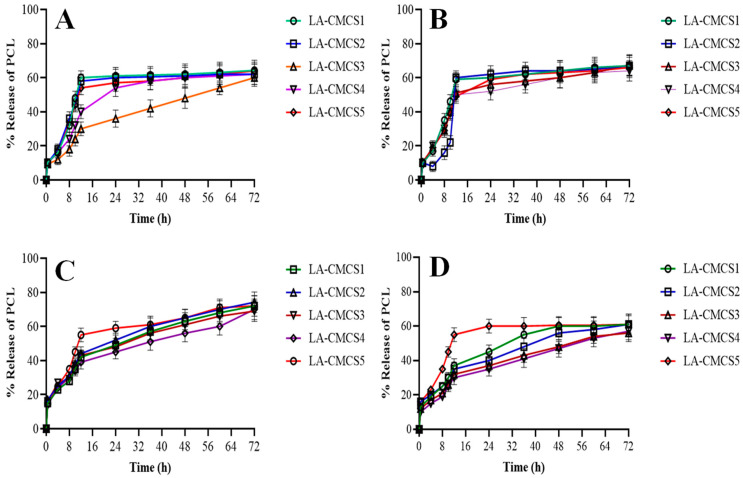
Release of PCL from LA-CMCS micelles in SGF with pepsin (**A**), SGF without pepsin (**B**), SIF with pancreatin (**C**), and SIF without pancreatin (**D**) for a period of 72 h.

The dialysis bag method was used to analyse the PCL in vitro release characteristics from the PCL-loaded LA-CMCS micelles in various release media. The release kinetics of the PCL-loaded LA-CMCS micelle were shown to be affected by the pH level of the release medium. The PCL-loaded LA-CMCS micelle exhibited a quicker release in the first 0.5 h, with about 10% of the PCL being rapidly released into acidic media of SGF pH 1.2 with or without pepsin, before reaching a plateau after 12 h. Just 67% of the encapsulated medication was released from micelles during a 72 h period after the initial burst release; micelles exhibited sustained release behaviour, as shown in Figure 3. Due to the increased protonation of the free amino groups of CMCS in the SGF medium (pH 1.2) and the released non-encapsulated drug part, the initial rapid release of the drug in an acidic environment may have been influenced by the creation of a loose micelle structure. Nevertheless, only roughly 64% of micelles in SGF with pepsin (pH 1.2) demonstrate a similar continuous release pattern over a 72 h period. In SIF without pancreatin (pH 6.8), the PCL-loaded LA-CMCS micelles showed delayed and persistent release characteristics of PCL, releasing only around 10% after 0.5 h, about 61% within 12 h, and then reaching a plateau over 24 h. Using pancreatin release media, the PCL-loaded LA-CMCS micelles exhibit burst release in SIF (pH 6.8); approximately 17% of the PCL was released within the first 0.5 h, and about 74% of the encapsulated PCL was released in the next 72 h. The development of loose aggregates brought on by the catalytic activity of amylase on CMCS in the presence of pancreatin is attributed to the initial burst release of PCL. The particle size at the end of the dissolution of all the prepared formulations was determined by the DLS method. LA-CMCS1 showed 78 nm and 75 nm sizes displayed by LA-CMCS2 formulation. LA-CMCS3, LA-CMCS4, and LA-CMCS showed 77, 82, and 80 nm, respectively. Table 3 displays the proposed profiles that were fitted according to practical release values as well as the estimated PCL release exponent (n) and rate constant (k). The micelles followed the kinetics of zero order with the Fickian diffusion mechanism as the value of *n* was less than 0.5. The value of *R*^2^ in zero order and first order ranged from 0.991 to 0.999 and 0.567 to 0.789, respectively, indicating the release of PCL from LA-CMCS micelles that followed first-order kinetics. The release behaviour for PCL-loaded LA-CMCS micelles was consistent with the Fickian diffusion when the n values were less than 0.5. The k values showed that drug-loaded LA-CMCS micelles released PCL gradually.

### 3.7. Stability of Micelles in Gastrointestinal Fluids

To simulate the gastrointestinal circumstances found in vivo, the GI stability of PCL-loaded LA-CMCS micelles was examined in a variety of dilution mediums, including SGF and SIF with or without enzymes. When the formulation is incubated in SGF without pepsin and SGF with pepsin enzyme for 2 h, the micelle size of PCL-loaded micelles exhibits minor changes. Because the unreacted amino groups of CMCS were protonated at an acidic pH, the cationic character of those groups caused the zeta potential of the micelle to shift from −29 ± 2 mV to +9 ± 3 mV, as shown in Table 4. However, after 6 h of incubation in SIF-containing pancreatin, the mean micelle size rose. This could be attributed to the amylase’s catalytic action on CMCS, which results in the creation of loose aggregates [34]. The PDI values of the micelles did not significantly deviate from their initial values during incubation in various media, indicating the stability of the micelles in gastrointestinal settings [35].

### 3.8. Storage Stability of Micelles

The size and PCL loading of the micelles were observed to assess their storage stability [36]. The micelles were stable in PBS at 4 °C for a minimum of four weeks. This was demonstrated by the minimal change in the particle size of the formulations and a less than 5% decrease in the PCL content in the micelle, as shown in Table 5. The particle size of the micelles in 50% serum was observed to assess the stability of the micelles in serum. The micelles size remained unchanged during a 24 h incubation period in serum.

### 3.9. Intestinal Permeability

**Figure 4:**
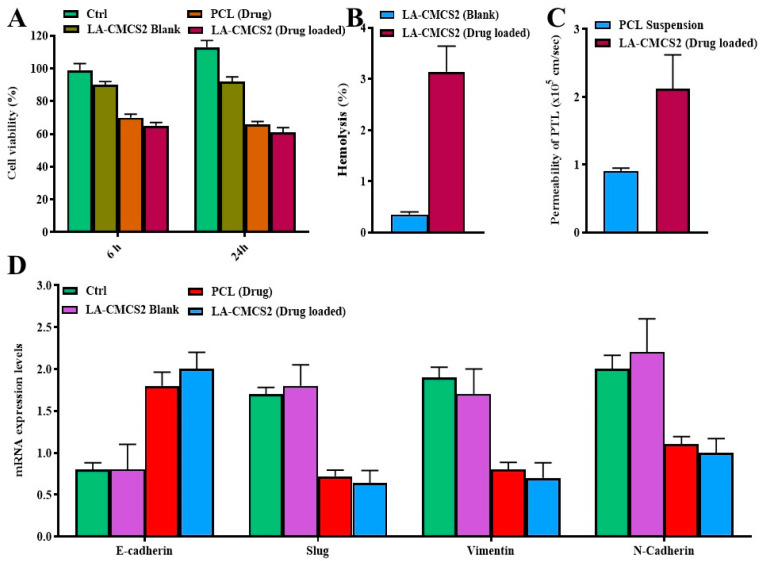
Biological studies of the micelles of LA-CMCS conjugates, cell viability (**A**), haemolysis test (**B**), permeability of PCL (**C**), and gene expression of epithelial and mesenchymal markers by qPCR, where epithelial marker like E-cadherin were found to be higher, while mesenchymal markers (Slug, Vimentin, and N-cadherin) were lower in cancer cells compared to the control (**D**).

This study evaluated the intestinal absorption of LA-CMCS2 nanoparticles by assessing the intestinal permeability of PCL in rat intestine segments. The LA-CMCS2 effective permeability of 2.36 × 10^−5^ cm/s was significantly (2.2 fold) higher than that of 0.94 ± 0.32 × 10^−5^ cm/s when compared to pure PCL suspension, showing that the micelles enhanced the intestinal absorption of PCL, as shown in Figure 4. Our findings are consistent with the results obtained by Qu et al. in 2018 for PCL’s intestinal permeability [37].

### 3.10. Cell Viability, qPCR, Western Blot, and Haemolysis Study

**Figure 5:**
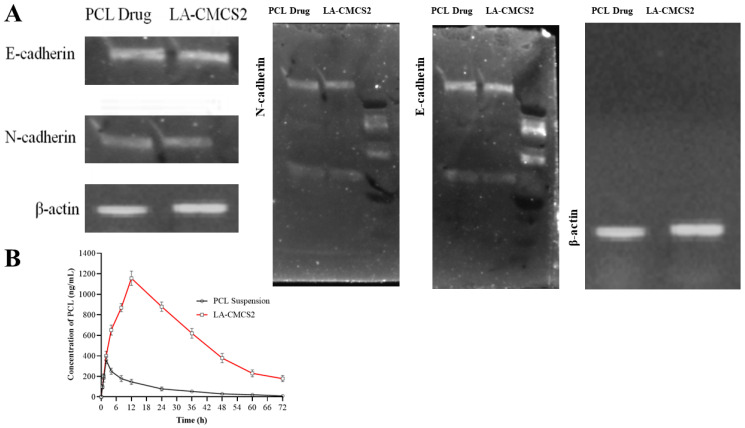
Western blot (**A**) and analysis of pharmacokinetics (**B**) of PCL from LA-CMCS2 and PCL suspension in albino rats.

The inhibition of Caco-2 cell proliferation was observed when these cells were incubated with LA-CMCS2 (drug-loaded)micelles, as depicted in Figure 4A. To ensure that the micelle formulation was not hazardous to red RBCs, an in vitro RBCs lysis test was conducted. A positive control with 100% RBC lysis was Triton X-100. LA-CMCS2 (drug-loaded) caused very minimal RBC lysis when compared to the blank micelle formulation LA-CMCS2, showing that they are appropriate for systemic delivery (Figure 4B). This is because nano-formulated water-soluble PCL reduces the side effects of haemolysis [38]. Chitosan-based nanocarriers can increase the paracellular permeability (Figure 4C) and thus enhance drug absorption [39]. Compared to PCL (drug), our micelle formulation demonstrated a considerable inhibitory effect on EMT, in which a significant upregulation of E-cadherin and downregulation of Slug, Vimentin, and N-cadherin were observed by real-time PCR, as shown in Figure 3D. Moreover, the protein expression of E-cadherin was upregulated, while that of N-cadherin was downregulated, as shown in Figure 5A.

### 3.11. Pharmacokinetic Study

The pharmacokinetic characteristics of experimental PCL-loaded LA-CMCS polymeric micelles were determined by using the HPLC validated method. For pure PCL and PCL-loaded micelles (LA-CMCS2), the plasma concentration and comparison with time are shown in Figure 5B and Table 6. Both profiles showed distinctive biphasic patterns; one phase revealed fast distribution, and the other one showed elimination. In the case of PCL suspension (Control), the time from 0 to 3 h showed the distribution phase, and the time from 4 to 72 h indicated the elimination phase. For PCL-loaded micelles (test formulation), the period from 0 to 12 h indicates the distribution phase, and the time from 12 to 72 h indicates the elimination phase of PCL from albino rats. The peak plasma concentrations from PCL suspension and LA-CMCS2 micelles were 3 and 12 h, respectively. The difference in time for the peak plasma concentration between the two formulations was due to the sustained release behaviour of PCL-loaded micelles. The release of PCL from micelles was sustained compared to the suspension of PCL. These findings demonstrate that the LA-CMCS2 micelle enhances PCL’s bioavailability and lowers its rate of excretion, which may boost its effectiveness. Different pharmacokinetic parameters of the micelles of LA-CMCS2 and pure PCL are displayed in Table 6. In both phases, distribution and elimination, the concentration of PCL contributed as the pure drug was lowered compared to PCL-loaded LA-CMCS2 micelles. The AUC_0–24_ value of LA-CMCS2 micelles was 3.3-fold greater than that of pure PCL. The plasma drug levels attained with paclitaxel alone were less than the limit of quantification (LOQ) of our HPLC assay. Therefore, the improvement in solubility offered by polymeric micelles is significant foraltering the plasma drug level. The AUC and C_max_ of the LA-CMCS2 group were considerably higher (*p* < 0.05) than those of the PCL suspension group (AUC = 3960 ng·h/mL, C_max_ = 360 ng/mL), with improvements of 3.32 and 3.21 times, respectively. The oral bioavailability of paclitaxel is undoubtedly enhanced by the improvement in solubility [40]. Additionally, it can be connected to increased permeability brought on by P-glycoprotein self-saturation as a result of a higher drug concentration at the gut lumen surface. Moreover, the PCL half-life (t_1/2_) increased by 3 to 6 times after being incorporated into LA-CMCS2 micelles. This could be the reason for the drug’ reduced clearance as polymeric micelles [41]. The absorption (t_1/2a_) and elimination (t_1/2b_)half-life of PCL suspension and LA-CMCS2 micelles were calculated, and they are outlined in Table 6. The developed micelles contain chitosan and linoleic acid, and these formulations underwent chylomicron formulation [35]. The chylomicron formed from fatty acid with chitosan is responsible for long-term circulation [36,37]. The developed formulations of LA-CMCS2 micelles might have followed a long circulation and release pattern. The plasma elimination rate constants unveiled by PCL from LA-CMCS2 micelles also revealed that the drugs may remain in systemic circulation for a long period of time. The results revealed that the absorption-related problems of PCL were successfully eliminated by making LA-CMCS2 polymeric micelles. Consequently, PCL-loaded LA-CMCS2 micelles may destroy tumour cells with adequate time retention, so PCL is considered to be efficient for use as an anticancer therapy.

### 3.12. Tissue Distribution Study

PCL concentrations were measured in the major organs (liver, spleen, kidney, heart, lung, and intestines) of rats after the oral administration of LA-CMCS2 micelles (1 mg/kg) and PCL suspension (1 mg/kg). Compared to the PCL suspension group, PCL was more broadly dispersed and concentrated in the tissues of the LA-CMCS2 group. Throughout the day, the PCL concentration in every organ examined in the LA-CMCS2 group was greater than the effective value (85.3 ng/mL) [42]. The concentration of PCL in the blood and heart was 3.22-fold and 3.17-fold higher in the LA-CMCS2 group compared to the PCL suspension group, respectively, as shown in Table 7. A 3.21- and 3.68-folds higher concentration of PCL in the LA-CMCS2 group was detected compared to the PCL suspension group in the lungs and kidneys, respectively. A similar pattern of a higher concentration of PCL from micelles in comparison to pure PCL was also observed in the liver and spleen of rats. In the stomach and colon, 2.96- and 5.30-times higher concentrations of PCL from LA-CMCS2 micelles were detected compared to the PCL suspension group.

## 4. Conclusions

LA-CMCS-conjugated micelles were developed to enhance the solubility and oral delivery of PCL. To encapsulate PCL and improve its oral bioavailability, we have developed amphiphilic conjugate-based polymeric micelles made of CMCS and LA in this study. By modifying CMCS with LA through an amidation process, LA-CMCS conjugate was developed, and it was characterised using spectroscopic methods. Furthermore, it showed the ability to self-assemble into micelle-like structures in an aqueous medium with ease. The PCL-loaded LA-CMCS micelles also showed remarkable stability in simulated body fluids with various pH levels. PCL-loaded LA-CMCS micelles had an extended release pattern in simulated bodily fluids according to an in vitro release investigation. Additionally, the findings of this permeation research showed that micelles made of polymers loaded with PCL exhibited enhanced intestinal absorption behaviour. Additionally, pharmacokinetic investigations revealed that when compared with a PCL suspension, LA-CMCS micelles with PCL loaded into them significantly improved oral absorption. Overall, this study concludes that amphiphilic CMCS analogues could serve as an effective drug delivery system for boosting the biopharmaceutical effectiveness of drugs with weak water solubility.

## Figures and Tables

**Table 1:** Size of micelles before and after drug loading, PDI, and zeta potential of developed formulations.

Code	Particle Size (nm) before Loading of Drug	Particle Size (nm) after Loading of Drug	PDI	Particle Size (nm) after Lyophilisation of PCL-Loaded Micelles	Zeta Potential (mV)
LA-CMCS1	99 ± 13	100 ± 18	0.198 ± 0.01	99.5 ± 13	−18 ± 2
LA-CMCS2	93 ± 11	94 ± 20	0.023 ± 0.01	94 ± 11	−29 ± 2
LA-CMCS3	101 ± 18	100 ± 21	0.208 ± 0.02	100.5 ± 14	−16 ± 2
LA-CMCS4	119 ± 12	121 ± 17	0.308 ± 0.03	120 ± 12	−24 ± 1
LA-CMCS5	108 ± 19	108 ± 21	0.287 ± 0.03	107.5 ± 15	−23 ± 3

**Table 2:** Values of %DL and %EE in micelles of various formulations.

Code	PCL:LA-CMCS	%DL	%EE
LA-CMCS1	1:2	65 ± 2	60 ± 2
LA-CMCS2	1:1	67 ± 3	61 ± 3
LA-CMCS3	1:4	64 ± 4	59 ± 2
LA-CMCS4	1:6	63 ± 3	57 ± 2
LA-CMCS5	1:8	62 ± 3	56 ± 4

**Table 3:** Values of kinetics of PCL release from LA-CMCS micelles.

Code	First Order	Zero Order	Higuchi	Korsmeyer–Peppas		
	*R* ^2^	*R* ^2^	*R* ^2^	*n*	*R* ^2^	*k*
LA-CMCS1	0.567	0.995	0.876	0.33	0.993	0.53
LA-CMCS2	0.598	0.999	0.898	0.21	0.998	0.49
LA-CMCS3	0.745	0.990	0.766	0.24	0.981	0.44
LA-CMCS4	0.609	0.991	0.821	0.38	0.989	0.42
LA-CMCS5	0.798	0.996	0.859	0.32	0.990	0.39

**Table 4:** Effects of various medium on the size, PII, and surface charge of LA-CMCS micelles.

Conditions	Size of Micelles (nm)	PDI	Surface Charge (mV)
SIF with pancreatin pH 6.8	108 ± 7	0.025 ± 0.02	+7 ± 1
SIF with pancreatin pH 6.8	107 ± 3	0.023 ± 0.03	+8 ± 2
SGF with pepsin pH 1.2	94 ± 5	0.024 ± 0.01	+7 ± 1
SGF without pepsin pH 1.2	94 ± 6	0.022 ± 0.02	+8 ± 2
PBS pH 5	99 ± 7	0.023 ± 0.03	+9 ± 3

**Table 5:** Results of storage stability studies.

Code	Size of Micelles after 4 Weeks	%DL of PCL after 4 Weeks
LA-CMCS1	99 ± 12	63 ± 3
LA-CMCS2	93 ± 07	65 ± 2
LA-CMCS3	98 ± 08	62 ± 3
LA-CMCS4	119 ± 13	61 ± 2
LA-CMCS5	106 ± 11	60 ± 3

**Table 6:** Pharmacokinetic parameters of PCL after oral administration of PCL suspension (control group) and micelles of LA-CMCS2 (test group).

Parameters	Units	PCL Suspension	LA-CMCS Micelles
C_max_	ng/mL	360 ± 86	1156 ± 18
T_max_	h	2 ± 1	12 ± 2
t_1/2_	h	9 ± 2	50 ± 10
t_1/2a_	h	10.82 ± 3	7.5 ± 3
t_1/2b_	h	15.4 ± 4	17.7 ± 4
AUC_0–t_	ng·h/mL	3960 ± 23	12,716 ± 78
AUC_0–ꝏ_	ng·h/mL	4312 ± 24	14,512 ± 82

**Table 7:** Concentration of PCL in different tissues of rats.

Samples	Concentration of PCL in ng/mL	
	LA-CMCS2 Micelles	Pure PCL
Blood	1158 ± 28	359 ± 46
Heart	2012 ± 31	634 ± 41
Lung	2324 ± 87	734 ± 32
Kidney	4532 ± 102	1231 ± 51
Liver	3345 ± 54	978 ± 59
Spleen	2139 ± 62	634 ± 21
Stomach	5234 ± 208	1765 ± 104
Jejunum	5039 ± 108	1345 ± 87
Ileum	9564 ± 234	1643 ± 106
Colon	9786 ± 298	1845 ± 107

## Data Availability

Data are contained within article and Supplementary Materials.

## Supplementary Materials

The following supporting information can be downloaded at: https://www.mdpi.com/article/10.3390/pharmaceutics16030342/s1, Figure S1: 1H-NMR of LA (A), CMCS (B), and LA-CMCS conjugate (C); Figure S2: Size of micelles of LC-CMCS2; Figure S3: Surface charge of micelles of LC-CMCS2; Figure S4: TEM image of LA-CMCS2-loaded micelles.
