# Supplementary material for: Conjugated Linoleic Acid–Carboxymethyl Chitosan Polymeric Micelles to Improve the Solubility and Oral Bioavailability of Paclitaxel
Source: Pharmaceutics. 2024 Feb 28;16(3):342. doi: 10.3390/pharmaceutics16030342 (PMC10974779; doi:10.3390/pharmaceutics16030342)
Supplement: Supplementary file 1 [file pharmaceutics-16-00342-s001.zip › pharmaceutics-2856699-supplementary.pdf]

Conjugated Linoleic Acid-Carboxymethyl Chitosan Polymeric Micelles to Improve the Solubility and Oral Bioavailability of Paclitaxel

Iqra Mubeen [1], Ghulam Abbas [1,*], Shahid Shah [2], Abdullah A Assiri [3]

[1]Department of Pharmaceutics, Faculty of Pharmaceutical Sciences, Government College University Faisalabad, Pakistan

[2]Department of Pharmacy Practice, Faculty of Pharmaceutical Sciences, Government College University Faisalabad, Pakistan

[3]Department of Clinical Pharmacy, College of Pharmacy, King Khalid University, Abha, Saudi Arabia

***Author for Correspondence**

*ghulamabbas@gcuf.edu.pk

1.1. ^{1}H-NMR of LA, CMCS and LA-CMCS conjugate

LA

[^{1}H-NMR, δ 5.46 (dtd, 1H), 5.39 – 5.30 (m, 1H), 2.58 (tp, 1H), 2.21 (t, 1H), 2.04 (tdd, 2H), 1.48 (p, 1H), 1.39 – 1.31 (m, 1H), 1.31 – 1.26 (m, 4H), 1.26 – 1.20 (m, 2H), 0.92 – 0.85 (m, 1H)].

CMCS

[^{1}H-NMR, δ 4.63 (dq, 1H), 4.33 (d, 1H), 4.15 (s, 2H), 3.94 (qq, 1H), 3.77 (qq, 1H), 3.73 – 3.62 (m, 2H), 3.35 (d, 3H), 3.19 (t, 1H), 3.15 – 3.03 (m, 2H), 1.87 (h, 1H), 1.03 (dt, 3H)].

LA-CMCS conjugate

[^{1} H-NMR, δ 7.35 (d, 1H), 5.54 – 5.45 (m, 2H), 5.39 – 5.30 (m, 2H), 4.77 (dq, 1H), 4.67 (d, 1H), 4.19 – 4.07 (m, 3H), 4.07 – 4.00 (m, 1H), 4.00 – 3.96 (m, 1H), 3.67 (d, 2H), 3.55 (dt, 1H), 3.47 (d, 3H), 2.52 (tdq, 2H), 2.18 (td, 2H), 2.04 (tdp, 4H), 1.94 (h, 1H), 1.51 (pd, 2H), 1.39 – 1.31 (m, 3H), 1.31 – 1.25 (m, 11H), 1.25 – 1.21 (m, 1H), 1.00 (dt, 3H), 0.94 – 0.84 (m, 3H)].

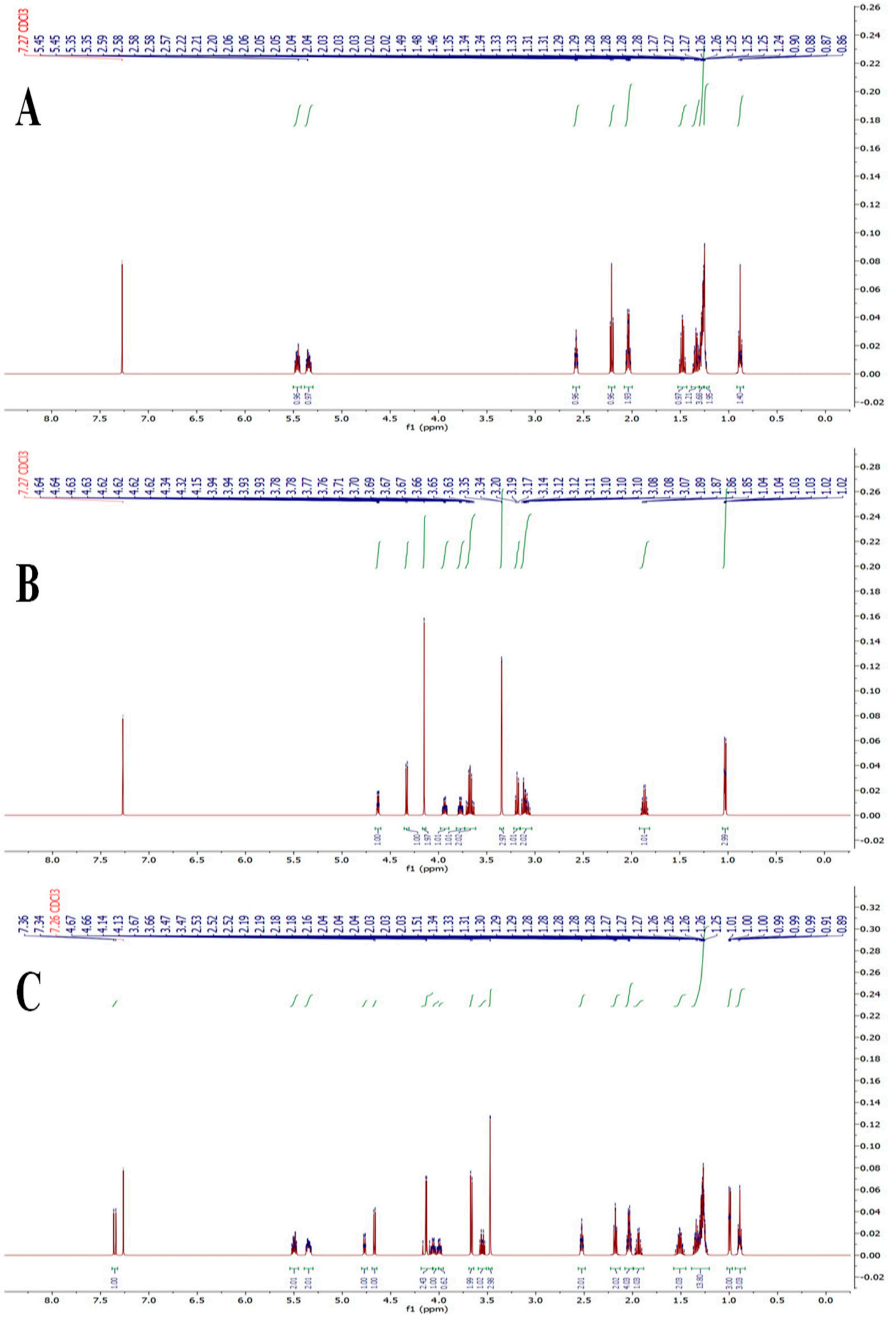

Figure S1: ^{1}H-NMR of LA (A), CMCS (B) and LA-CMCS conjugate (C)

1.2. Particle size and zeta potential analysis of LA-CMCS2 micelles

Results

			Size (d.nm):	% Intensity:	St Dev (d.n...
**Z-Average (d.nm):**	93.0	**Peak 1:**	93.0	100.0	19.85
**PdI:**	0.023	**Peak 2:**	0.000	0.0	0.000
**Intercept:**	0.843	**Peak 3:**	0.000	0.0	0.000
**Result quality :**	**Good**				

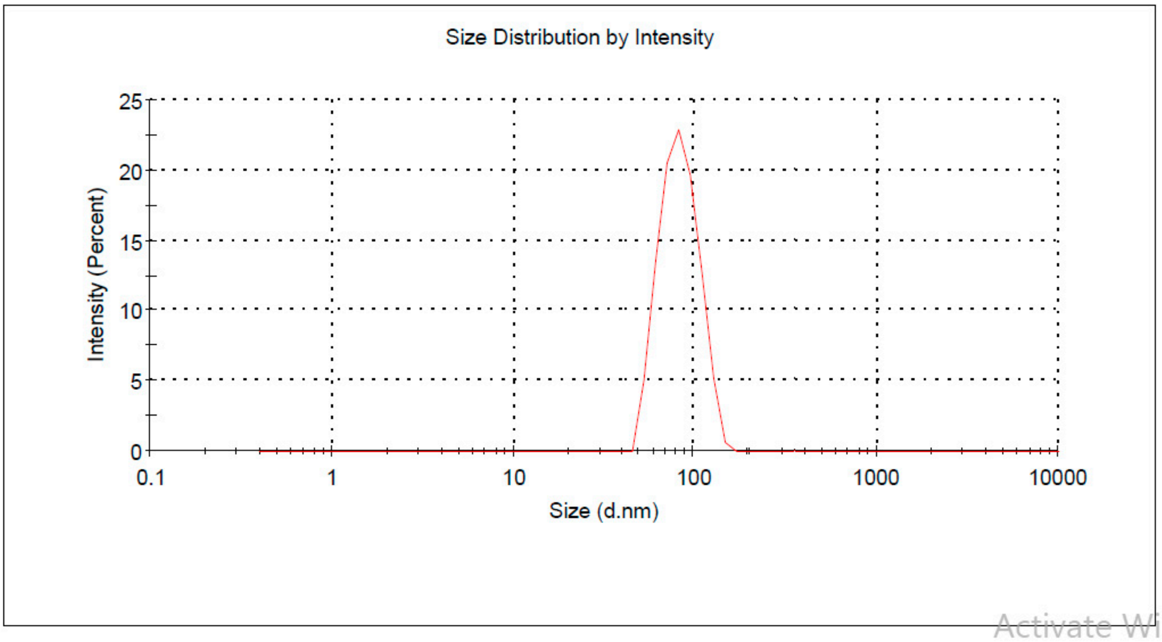

Activate Win

Figure S2: Size of micelles of LC-CMCS2

Results

			Mean (mV)	Area (%)	St Dev (mV)
**Zeta Potential (mV):**	-29.0	**Peak 1:**	-29.0	100.0	5.18
**Zeta Deviation (mV):**	5.18	**Peak 2:**	0.00	0.0	0.00
**Conductivity (mS/cm):**	0.0159	**Peak 3:**	0.00	0.0	0.00
**Result quality :**	See result quality report				

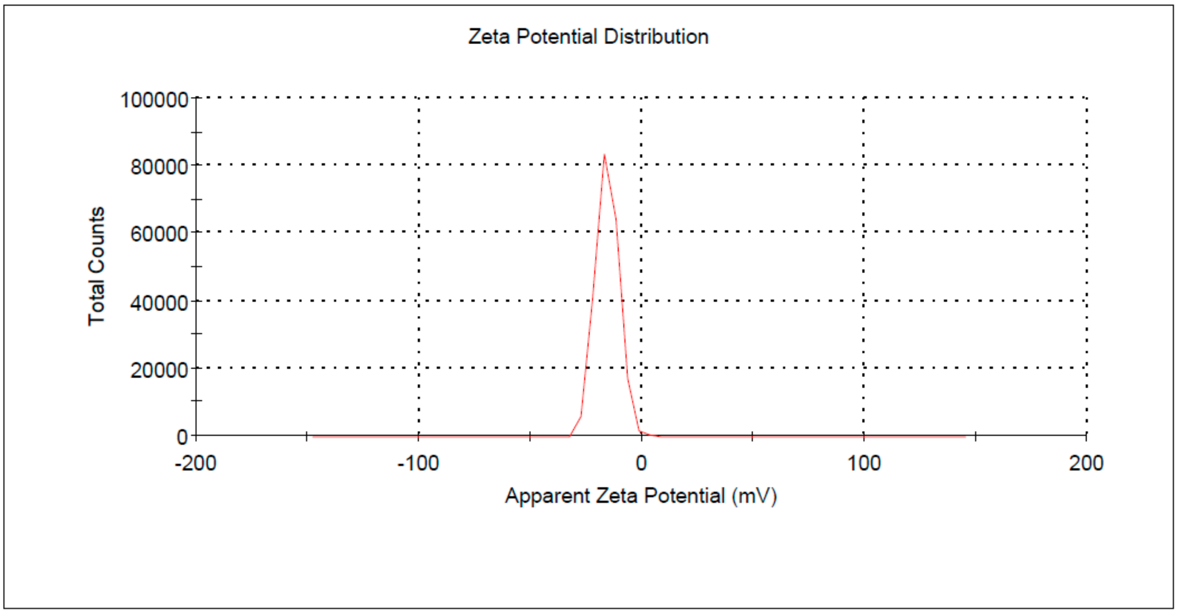

Figure S3: Surface charge of micelles of LC-CMCS2

1.3. Transmission electron microscopy (TEM)

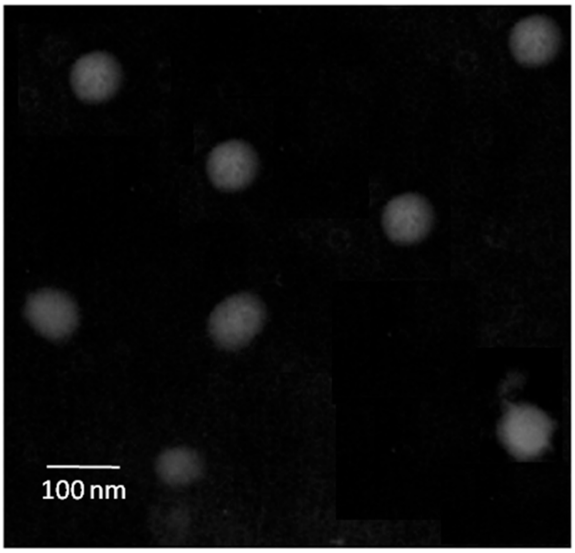

Figure S4: TEM image of LA-CMCS2 drug loaded micelles
